# Expression, purification and biochemical characterization of *Schizosaccharomyces pombe* Mcm4, 6 and 7

**DOI:** 10.1186/1471-2091-14-5

**Published:** 2013-02-27

**Authors:** Meng Xu, Y Paul Chang, Xiaojiang S Chen

**Affiliations:** 1 Graduate Program in Genetics, Molecular and Cell Biology, University of Southern California, Los Angeles, CA, 90089, USA; 2 Molecular and Computational Biology Program, Departments of Biological Sciences/Chemistry, University of Southern California, Los Angeles, CA, 90089, USA; 3 Norris Cancer Center, University of Southern California, Los Angeles, CA, 90089, USA

**Keywords:** Cell cycle proteins, DNA-binding proteins, Recombinant proteins, Protein binding, Protein oligomerization, *Schizosaccharomyces pombe*, *Escherichiai coli*

## Abstract

**Background:**

The hetero-hexamer of the eukaryotic minichromosome maintenance (MCM) proteins plays an essential role in replication of genomic DNA. The ring-shaped Mcm2-7 hexamers comprising one of each subunit show helicase activity *in vitro*, and form double-hexamers on DNA. The Mcm4/6/7 also forms a hexameric complex with helicase activity *in vitro*.

**Results:**

We used an *Escherichiai coli* expression system to express various domains of *Schizosaccharomyces pombe* Mcm4, 6 and 7 in order to characterize their domain structure, oligomeric states, and possible inter-/intra-subunit interactions. We also successfully employed a co-expression system to express Mcm4/6/7 at the same time in *Escherichiai coli*, and have purified functional Mcm4/6/7 complex in a hexameric state in high yield and purity, providing a means for generating large quantity of proteins for future structural and biochemical studies.

**Conclusions:**

Based on our results and those of others, models were proposed for the subunit arrangement and architecture of both the Mcm4/6/7 hexamer and the Mcm2-7 double-hexamer.

## Background

Within the MCM family, Mcm2-7 proteins are revealed as key components of the pre-replicative complex (pre-RC). Pre-RC initiates DNA synthesis at the origin in all eukaryotes [1-3]. Mcm2-7 are six proteins that are homologous to each other and are conserved among Archaea and eukaryotes [4]. Mcm2-7 functions as the replicative helicase, and can form various oligomeric complexes, including double-hexamers [5,6], hexamers [7,8], tetramers [9], trimers [10], and dimers [7,11,12].

It has been well demonstrated that Mcm2-7 are vital in the initiation and the elongation of genomic DNA replication as a eukaryotic replicative helicase. Purified Mcm2-7 hexamer has helicase activities *in vitro* if glutamate is included in the reaction buffers [13]. In addition, helicase activity has been shown *in vitro* for MCM sub-complex comprising only three of the six subunits, Mcm4/6/7 hexamers (two copies of each subunit).

To further understand the subunit arrangement and architecture of the Mcm4/6/7 hexamer assembly, we characterized individual domains and near-full-length polypeptides of each of subunits using *E. coli* expression. Various truncated fragments of *Schizosaccharomyces pombe* Mcm4, 6 and 7 were purified, and then their oligomeric states and inter-subunit interactions were investigated *in vitro* by gel filtration and pull-down assays. By using a co-expression system developed in *E. coli*, we successfully purified in large quantity of soluble and pure *S. pombe* Mcm4/6/7 complex in hexameric state.

## Methods

### Reagents

Oligonucleotides were synthesized by Integrated DNA Technologies (IDT) or Eurofins MWG Operon. Pfu Turbo polymerase was purchased from Stratagene. Ni-NTA affinity resin is purchased from QIAGEN. pGEX-6P-1 vector, PreScission protease, Glutathione affinity column, Resource Q column, Superdex 200 and Superose 6 10/300 GL gel filtration column were purchased from GE Healthcare Biosciences Amersham. The pXA/BN-based vectors, used for protein co-expression, were engineered from the original pAC vector described [14]. PMSF is purchased from Sigma-Aldrich.

### MCM fragments designs and plasmid construction

To design various spMcm fragments, native disorder in proteins is determined by the DISOPRED server at University College London [15]. Secondary structure prediction was performed on the PSIPRED server at University College London [16,17]. To determine the precise boundaries of the fragments, conserved amino acid residues were identified by protein sequence alignment among MCM proteins from various organisms (Additional file 1: Figure S1). Structural alignment to solved MCM structures was also conducted [18]. The multiple sequence alignment was performed using ClustalX [19].

**Figure 1:**
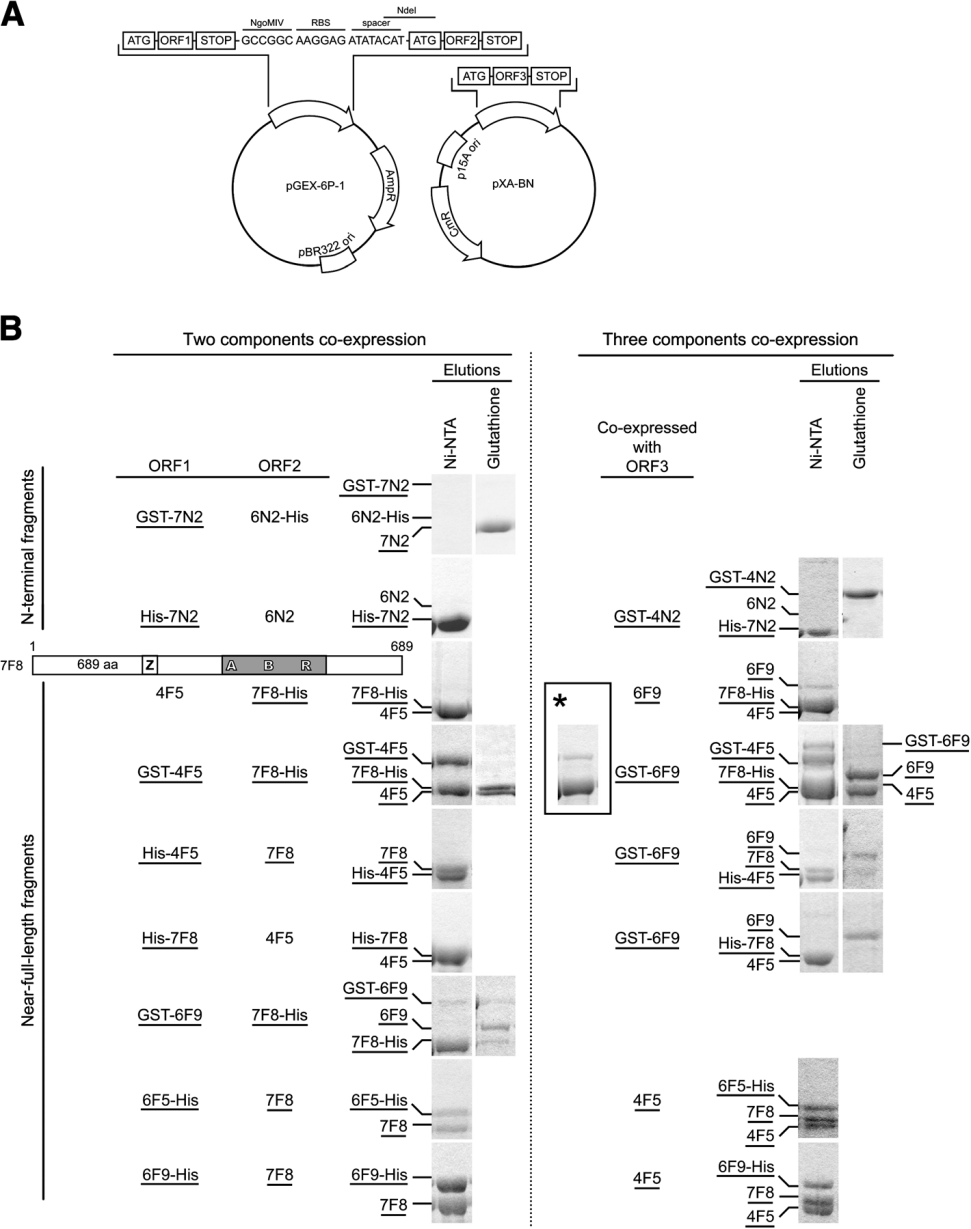
**Interactions and oligomeric states of co-expressed fragments of Mcm4, 6 and 7.** (**A**) Schematic of the polycistronic co-expression strategy that involves two compatible vectors. ORF1 and ORF2 were linked by a ribosome binding site (RBS) with a spacer. ORF3 was cloned in pXA-BN vector. Two plasmids were co-transformed into *E. coli*., followed by dual screening of ampicillin (50 μg/ml) and chloramphenicol (17 μg/ml). (**B**) Interactions of co-expressed and copurified fragments of Mcm4, 6 and 7, as identified in the two components co-expression (left side) or three components co-expression (right side) experiments. *E. coli.* lysates co-expressing various fragments with or without tags were passed through either glutathione or Ni-NTA resins, then the resins were washed as described under “Materials and Methods”. GST tags were cleaved by PreScission protease on the resin to release the MCM proteins. His tagged proteins were eluted by imidazole. All elutions were analyzed by SDS-PAGE. *Asterisk* denotes the co-lysis (instead of co-expression) of the indicated near-full-length fragments.

DNAs containing cDNA fragments encoding full length *spMCM 4* (GenBank:P29458), *6* (GenBank:CAB75412) and *7* (GenBank:O75001) (generously provided by Dr. J. Hurwitz, Memorial Sloan-Kettering Cancer Center, United States) were used as template in PCR with Pfu Turbo polymerase to obtain amplified coding sequences of various fragments. cDNA of N-terminal GST tagged fragments were subcloned to the NheI-AscI sites of pGEX-6P-1 or the NgoMIV-AscI sites of pXA-BN. cDNAs of N-terminal His Tagged fragments were subcloned to the NheI-AscI sites of pGEX-6P-1 with cDNA of GST removed. For co-expression (Figure 1A), ORF1s were subcloned to the NheI-NgoMIV sites followed by ORF2s to the NdeI-AscI sites, on pGEX-6P-1; ORF3s were subcloned to the NgoMIV-AscI sties of pXA-BN.

### Expression and purification of the fragments of Mcm4, 6 and 7

For the expression of various fragments of Mcm4, 6 and 7, constructs expressing each spMcm4, 6 and 7 fragments were transformed into *E. coli* by electroporation. Then the expression of proteins was induced by adding IPTG to 2 mM at 18°C when the cell density reached OD ~ 0.6. After cells were lysed by French Press, GST and His tagged fragments were purified by glutathione and Ni-NTA affinity chromatography, respectively. For GST tagged fragments, GST tags were subsequently removed by PreScission protease treatment in standard lysis buffer containing 250 mM NaCl, 50 mM Tris pH8 (buffer A) and 1 mM DTT. For His tagged fragments, buffer A containing 5 mM β-mercaptoethanol was used to lysate cell pellets and buffer A containing 5 mM β-mercaptoethanol and 100 ~ 150 mM imidazole was used for elution. The elution was loaded to a Superdex 200 or Superose 6 gel filtration column that is equilibrated with buffer A containing 1 mM DTT to finish the purification.

### Co-expression and copurification of near-full-length fragments of Mcm4, 6, and 7

The near-full-length (nFL hereafter) fragments of Mcm4, 6, and 7 were cloned into two compatible vectors (pGEX-6P-1 and pXA-BN) and co-expressed in *E. coli* (Figure 1A). Dual screening of ampicillin (50 μg/ml) and chloramphenicol (17 μg/ml) was used to maintain the stable expression. Then co-purification was conducted the same as described for individual fragments of Mcm4, 6, and 7. For the Mcm4/6/7 complex purification, cell pellets were resuspended and lysed in buffer A containing 5 mM β-mercaptoethanol. PMSF is added to 1 mM to prevent degradation. The supernatant from the lysis was passed through a Ni-NTA resin column. After extensive wash (10 × column volume) of the resin with buffer A containing 5 mM β-mercaptoethanol, the Mcm4/6/7 complex bound to the column through the C-terminal 8xHis tagged Mcm6 nFL was eluted by imidazole (150 mM). The eluted proteins were further purified using Resource Q anion-exchange chromatography with a 50 to 1000 mM NaCl gradient elution, followed by gel filtration chromatography with a Superdex-200 column that was pre-equilibrated with buffer A and 1 mM DTT. The proteins from the hexamer peak fractions were analyzed by SDS-PAGE and concentrated to ~50 mg/ml.

### Gel filtration analysis

A portion of the purified fraction (Glutathione affinity column eluate, 100 ~ 500 μg) was loaded to an analytical Superdex 200 or Superose 6 gel filtration column that is equilibrated with buffer A and 1 mM DTT. Fractions were collected and analyzed for composition by SDS PAGE and then staining with Coomassie brilliant blue (R250).

### Heilicase assay

Helicase assay was performed as described [20]. To obtain the dsDNA substrate, ~10 fmol of [γ-^32^P]-ATP ssDNA (60nt) was annealed to the circular M13mp18 ssDNA. The complementary sequence is 35nt, leaving a 25nt 5^′^ overhang on the substrate. Labeled substrate DNA was incubated with 100 ~ 200 ng Mcm4/6/7 hexamer in helicase buffer containing 25 mM Hepes pH7.5, 10 mM magnesium acetate, 5 mM ATP, 1 mM DTT and 0.1 mg/ml BSA for 45 min at 37°C. The reaction was analyzed on 12% native polyacrylamide gel. The gels were then dried and autoradiographed.

## Results

### Designs of truncated fragments of Mcm4, 6 and 7

**Figure 2:**
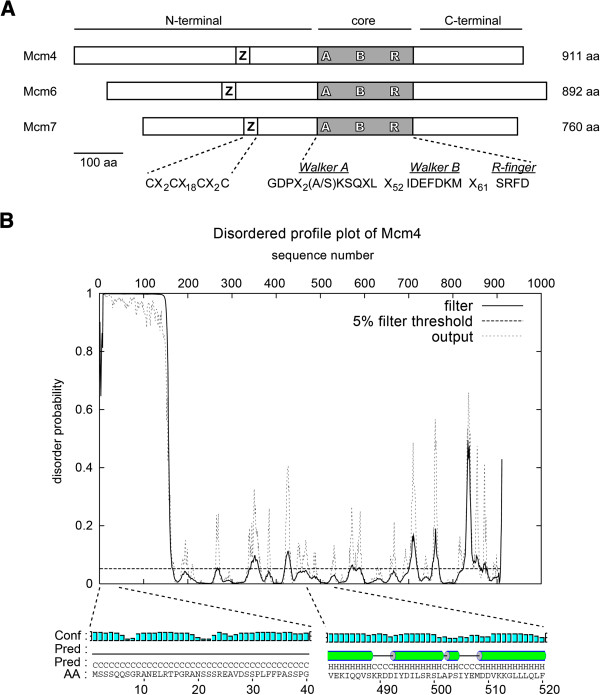
**Designs of truncated fragments of Mcm4, 6 and 7.** (**A**) Schematic of fission yeast Mcm4, 6 and 7. Locations of putative zinc finger (white boxes labeled with Z), the MCM core region (gray boxes) was shown. Three ATPase consensus motifs in the MCM core region were labeled with A (the Walker A motif), B (the Walker B motif) and R (the Arg-finger motif). All conversed amino acid residues that define each motif were shown. All truncation fragments reported in this paper were designed according to three domains, N-terminal, core and C-terminal domains. This figure was generated from the sequence alignment results shown in Additional File 1: Figure S1 and each Mcm protein was aligned with the MCM box region. (**B**) Disordered profile plot and predicted secondary structure of Mcm4. Only sampled secondary structure prediction was shown and aligned with the disordered profile. A disordered N-termini was present and aligned well with a region (1–150 aa) that lacks any defined secondary structure, while regions with very low disorder probability were predicted to show ordered secondary structures. The disordered profiles were generated by the DISOPRED server, and secondary structure prediction was generated by the PSIPRED server at University College London [15-17]. “Conf”-prediction confidence, “Pred”-predicted secondary structures, “AA”-amino acid residues. Disordered profile plots of Mcm6 and 7 were shown in Additional file 2: Figure S2.

To get stably expressed and soluble constructs of Mcm4, 6 and 7 in *E. coli*, it is important to make truncations around disordered regions or less conserved areas, but not in highly conserved and well folded regions. We first predicted the disordered parts of the native proteins using the DISOPRED server at University College London (Figure 2B, Additional file 1: Figure S1). The secondary structures of Mcm4, 6 and 7 were predicted using the PSIPRED server at University College London (Figure 2B, Additional file 2: Figure S2). In addition, we also performed structural alignments and comparison using solved archaeal MCM structures, such as structures of *Methanothermobacter thermautotrophicus* MCM (MtMCM, PDB:1LTL) and *Sulfolobus solfataricus* MCM (SsoMCM, PDB:3F9V), to more precisely determine the appropriate boundaries of the predicted secondary structures [18,21]. These results form the basis for deciding where to make truncations/deletions for protein expression.

**Figure 3:**
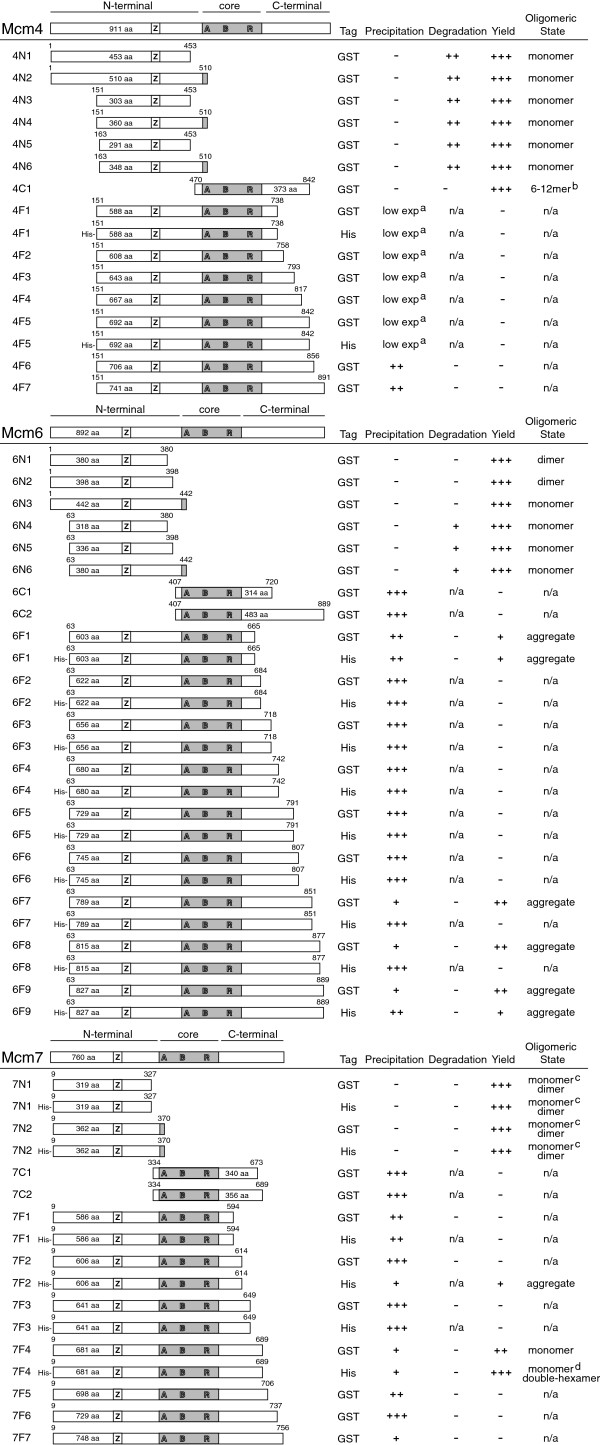
**Summary of biochemical properties of fragments of Mcm4, 6 and 7.** Schematic of truncated fragments of Mcm4, 6 and 7 tested in this study. The motifs are represented by: “A”-Walker A motif, “B”-Walker B motif, “R”-Arg-finger motif, “Z”-zinc finger motif. The nomenclature for the fragments is as follows, the first numbers represent the Mcm 4, 6, or 7; the letters in the middle indicate domain locations (“N”-N terminal fragments, “C”-core fragments, “F”-near-full-length fragments); the last numbers denotes construct number. a, decreased expression level or plasmid instability; b, oligomeric states depended on protein concentration; c, little equilibrium between monomeric and dimeric states and proteins in the two states could be separated by ion-exchange chromatography; d, a stable large complex identified with a molecular weight equal to a double-hexamer; n/a, not available, due to lack of enough samples.

We made three major MCM constructs groups in this study, N-terminal fragments, MCM core fragments, and the nFL fragments. The summary of the constructs and the observed biochemical properties were shown in Figure 3.

### Purification and characterization of N-terminal fragments of Mcm4, 6 and 7

**Figure 4:**
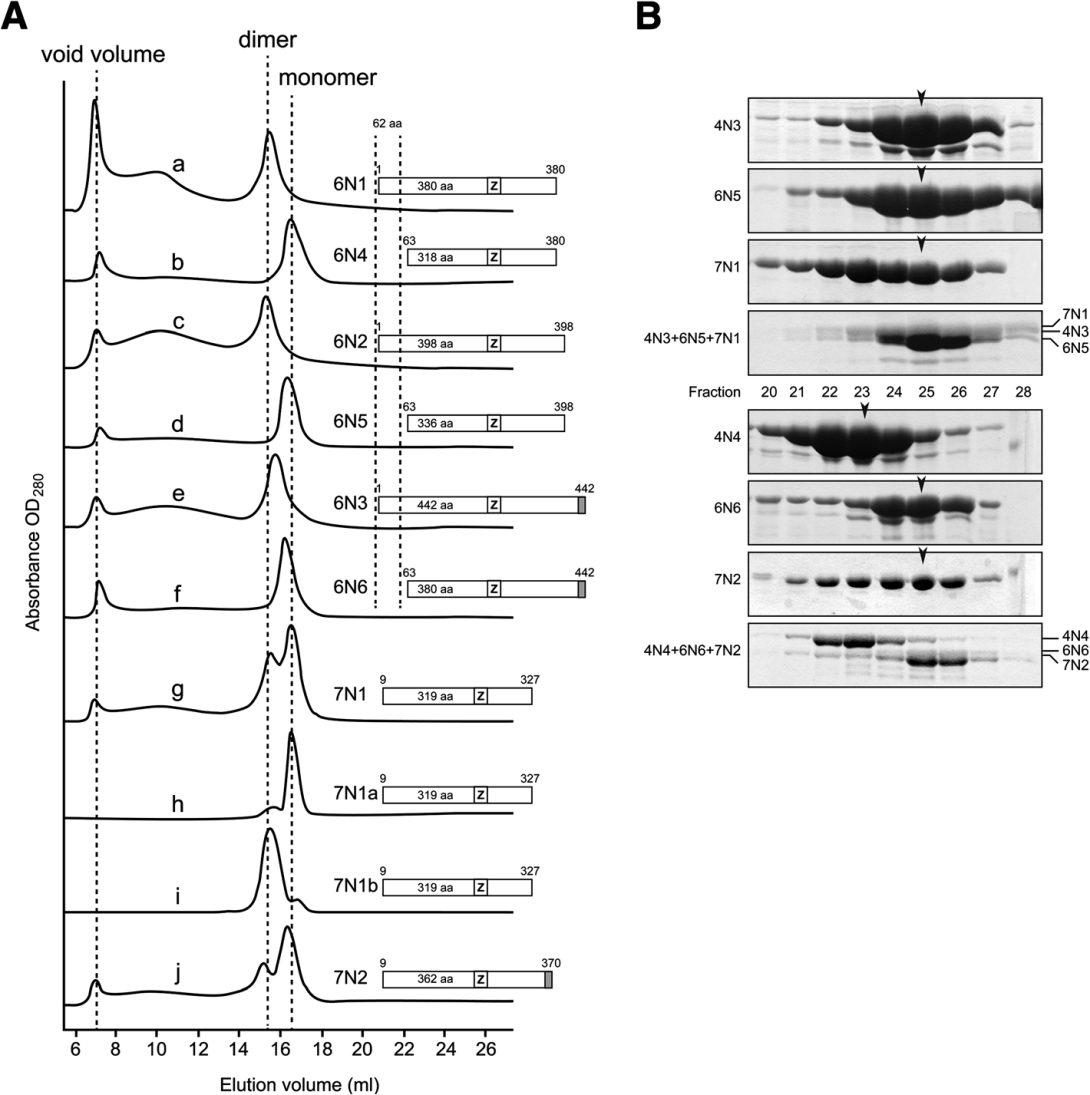
**Oligomeric states and interactions of N-terminal fragments of Mcm4, 6 and 7.** (**A**) Gel filtration chromatography profiles of N-terminal fragments of Mcm4, 6 and 7. Schematic of each fragment was shown in accordance with its gel filtration profile. N-terminal fragments of Mcm6 were aligned with the zinc finger motif and a 62 amino acid residues protruding N-termini was shown. 7N1a, separated monomeric 7 N1 fragment; 7N1b, separated dimeric 7 N1 fragment. Gel filtration analysis was carried out a described under “Materials and Methods”. (**B**) *In vitro* incubation of purified N-terminal fragments of Mcm4, 6 and 7. Interactions among the N-terminal fragments of Mcm4, 6 and 7 were characterized by gel filtration analysis. Samples from peak fractions (pointed by arrows) were quantitated by SDS-PAGE and mixed together in approximate equal molar ratio. The mixture was buffer-exchanged to 50 mM NaCl, 50 mM Tris pH8 and 1 mM DTT and then incubated on ice for 30 minutes. For 7 N1 and 7 N2, only samples from peak fraction of monomeric states were used. The incubation mixtures were subjected to gel filtration analysis and no large complex was detected. Two groups of N-terminal fragments of Mcm4, 6 and 7 were used, as shown in top and bottom panels.

Because the N-terminal fragment of MtMCM and SsoMCM oligomerize into hexamers [18,22], we want to investigate the role of the N-terminal fragments of Mcm4/6/7 in modulating oligomerization. Analysis of the purified proteins by gel filtration chromatography showed that most of the N-terminal fragments behaved as monomers (Figure 3). However for some N-terminal fragments of Mcm6 and Mcm7, peaks corresponding to a dimer formation were observed. As shown in Figure 4A, two out of three Mcm6 N-terminal fragments with intact N-terminus, 6 N1 and 6 N2, formed single peaks at the dimer position on gel filtration profiles. In contrast, the other three N-terminal fragments with N-terminal truncation, 6 N4, 6 N5 and 6 N6, only had peaks at monomer position.

For Mcm7 N-terminal fragments, 7 N1 and 7 N2, they showed two oligomeric peaks at the positions expected for dimers and monomers (Figure 4Ag and j). The fact that the two oligomeric states could be separated by Resource Q anion-exchange chromatography showed there was little equilibrium between the monomeric and dimeric states (Figure 4Ah and i).

To test whether the N-terminal fragments of Mcm4, 6 and 7 are competent to form hetero-oligomers, several combinations of the N-terminal fragments from Mcm4/6/7 were incubated together after purified individually. A relatively low salt concentration (50 mM NaCl) was used to favor oligomerization. However, no oligomer was identified under our tested conditions (Figure 4B).

### Purification and characterization of core fragments of Mcm4, 6 and 7

**Figure 5:**
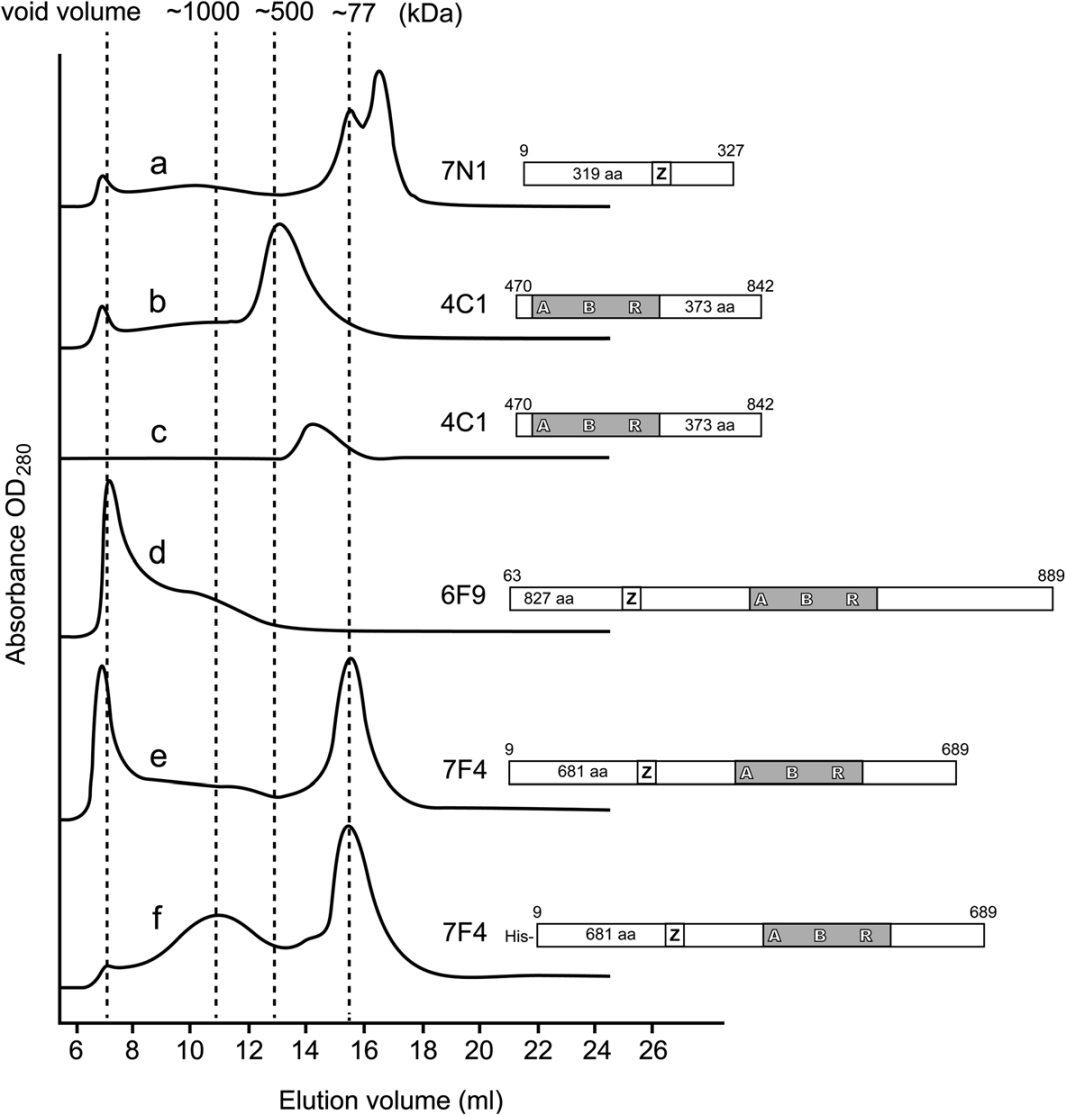
**Gel filtration chromatography profiles of core and near-full-length fragments of Mcm4, 6 and 7.** Schematic of each fragment was shown in accordance with its gel filtration profile. Gel filtration analysis was carried out a described under “Methods”. (**a**) Gel filtration profile of 7 N1 was chosen as a reference, and its dimer peak was used to align with monomer peaks of 7 F4. The other molecular weight shown was determined by Bio-Rad Gel Filtration Standard (data not shown). (**b-c**) Concentration dependent oligomerization of a core fragment of Mcm4, 4C1. **(d)** Large and heterogeneous aggregates composed of a nFL fragment of Mcm6, 6 F9. (**e-f**) Two oligomeric states of a nFL fragment of Mcm7, 7 F4. Peaks on the gel filtration profile correspond to the monomer and the double-hexamer.

Most of core fragment constructs of Mcm4, 6 and 7 suffered from heavy precipitation and only soluble protein of one fragment, 4C1, could be obtained (Figure 3). The oligomeric states of this fragment appeared at peaks with ~250 or 500 kDa, respectively in agreement with hexamers and 12-mers, depending on the protein concentration (Figure 5c and b). When a center fraction of the 12-mer peak was injected to the same gel filtration column, a hexamer peak appeared (Figure 5c), indicating that the two oligomeric states can equilibrate with each other. The protein concentration for the hexamer peak is much lower, compared to that of the 12-mer peak. Addition of ATP and Mg^2+^ did not affect the transition between the two oligomeric forms.

### Purification and characterization of nFL of Mcm4, 6 and 7

To help with purification, nFL fragments of Mcm4, 6 and 7 were tagged with GST or 8xHis and expressed in *E. coli*. In contrast to N-terminal fragments, these 70 ~ 90 kDa fragments were either insoluble or degraded when expressed in *E. coli*. Only one nFL fragment of Mcm7, 7 F4, could be successfully expressed and purified. We also found that the oligomeric states of this fragment changed when different N-terminal tags were used. As shown in Figure 5f, His tagged 7 F4 can form a very large and broad complex peak (about 1000 kDa) and a monomer peak. The large complex peak of His tagged 7 F4 was quite stable even at 1 M NaCl. In comparison, the same 7 F4 fragment that was cleaved from the GST-7 F4 fusion only appeared as in monomeric state (Figure 5e), suggesting the N-terminal GST tag may influence the self-interaction of this fragment.

As for nFL Mcm6 fragments, most of them precipitated in the cell pellets. Fragment 6 F9 could be purified but formed aggregates (Figure 5d). All nFL Mcm4 fragments had very low expression level. For 4 F5, the expressed protein seemed to be toxic to *E. coli* cells as the plasmid was instable (data not shown).

### Co-expression, copurification and characterization of complexes of Mcm4, 6 and 7

Because the nFL fragments of individual Mcm4, 6, and 7 expressed in *E. coli* did not behave well, we tried co-expression of all three proteins together to see if any stable complexes of them can be obtained. A polycistronic strategy (Figure 1A) using two compatible vectors was employed to co-express Mcm4, 6 and 7 in the same host cells. Various combinations of constructs were tested and the results were summarized in Figure 1B. A series of pull-down assays was also performed with either Ni-NTA or glutathione resin. It should be noted that the GST tag had been removed by PreScission protease in the elution, while either N-terminal or C-terminal 8xHis tag still remained.

As shown in Figure 1B, not all ORFs were translated, as in the cases of 6 N2-His, 6 N2, and 4 F5. A new nFL fragment of Mcm7, 7 F8, which includes an untruncated N-terminus, was used. As for the N-terminal fragments, no interactions between 4 N2 and 7 N2 was observed, given the negative reciprocal pull-down results.

**Figure 6:**
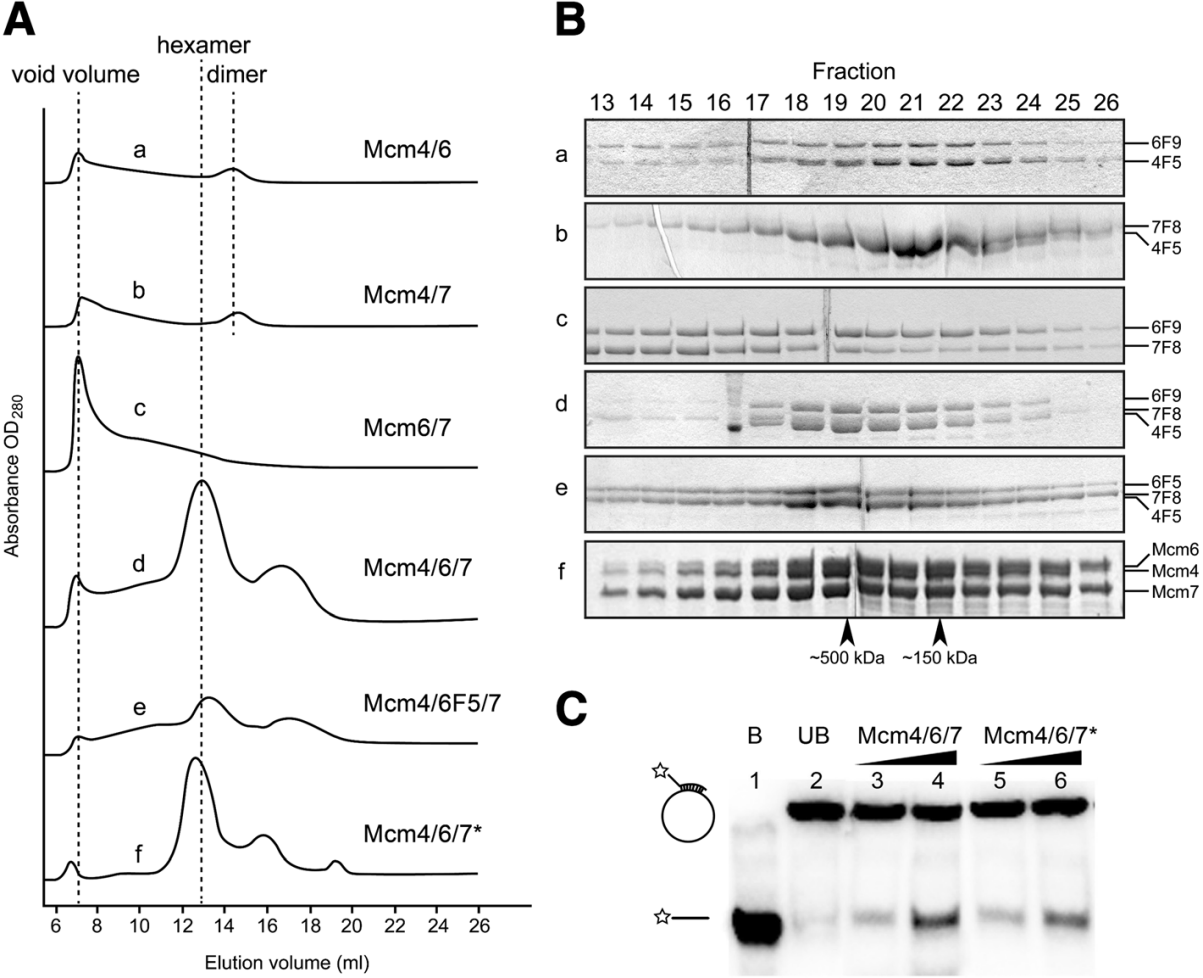
**Identification of stable Mcm complexes and helicase activity of Mcm4/6/7 hexamers.** (**A**) Gel filtration chromatography profiles of purified complexes of Mcm4, 6 and 7. Gel filtration analysis was carried out a described under “Materials and Methods”. *Asterisk*: Gel filtration profile of Mcm4/6/7 hexamers expressed and purified from insect cells in our laboratory. (**B**) SDS-PAGE analysis of purified complexes of Mcm4, 6 and 7 from the gel filtration fractions shown in Panel C. (**C**) Helicase assay results of the Mcm4/6/7 hexamers. No protein added in lane1 and 2. B, boiled substrate; UB, un-boiled substrate; lane 3 and 5, 100 ng protein added; lane 4 and 6, 200 ng protein added; *Asterisk*, Mcm4/6/7 hexamers expressed and purified from insect cells.

For the nFL fragments, strong interactions of 4 F5/6 F9, 4 F5/7 F8 and 6 F9/7 F8 were characterized by positive pull-down results. Most positive pull-down results were verified in two directions and showed little difference no matter which fragments was tagged, except 6 F9/7 F8 pair. When 7 F8 was tagged and used to pull-down 6 F9, only a weak interaction was detected, indicated by a very faint band of 6 F9. 1:1 molar stoichiometry of those binding pairs was also shown by SDS-PAGE analysis. Further gel filtration analysis clearly showed dimer peaks of 4 F5/6 F9 and 4 F5/7 F8 (Figure 6Aa and b), whereas only aggregates were observed on gel filtration profile of 6 F9/7 F8 (Figure 6Ac). Fractions obtained from gel filtration analysis were characterized by SDS-PAGE analysis, as shown in Figure 6B. Several co-expression combinations were able to produce all three nFL fragments of Mcm4, 6 and 7, however, combinations with N-terminal GST tagged 6 F9 still suffered from poor folding, which was implied by its very low yield and background binding with Mcm4 and 7 fragments.

We also compared co-expression results to co-lysis results. In co-lysis, GST-6 F9, GST-4 F5 and 7 F8-His were expressed individually, and cell pellets of their host cells were lysed together to provide binding environment similar to intracellular condition. As shown in Figure 1B *Asterisk*, when 7 F8-His was purified by Ni-NTA resin, only a small amount of GST-4 F5 were co-pulled down, and none of GST-6 F9 could be co-pulled down. In contrast, when these fragments were co-expressed, much stronger bindings were identified, indicating improved folding of these fragments. However, no hexamer could be purified when each protein was expressed separately first and the cells of each were co-lysed and incubated together, indicating that co-expression is needed for stable complex formation.

pGEX-6 F9-His-7 F8/pXA-4 F5 eventually produced the Mcm4/6/7 hexamer with a yield of 10 mg from 12 L culture. 8xHis tag was tagged on C-terminal of the Mcm6 fragment, and the Mcm4 and 7 fragments were not tagged. The three nFL fragments were co-expressed and copurified with a Ni-NTA affinity column that was able to bind 8xHis tags on the Mcm6 fragment. The Mcm4 and Mcm7 fragments were co-pulled down, indicating strong bindings among the three subunits. The complexes showed a single peak of about 500 kDa on gel filtration profiles, which is equivalent to the theoretical molecular weight (497 kDa) of the hexamer, consisting of the nFL fragments of Mcm4, 6 and 7 (Figure 6Ad). The size of the peak was also verified by aligning with the hexamer peak composed of Mcm4, 6 and 7 purified from insect cells (Figure 6Af).

A 1:1:1 molar stoichiometry for three proteins was shown in Figure 6 Bd. The central fraction of the peak was sent to N-terminal sequencing to confirm that the complex was composed of the Mcm4, 6 and 7 fragments. The salt resistance of the hexamer was also tested in various concentrations of NaCl ranging from 50 to 1000 mM, and appeared as a stably assembled oligomer that is suitable for further studies (data not shown). The final purified Mcm4, 6 and 7 hexamer could be concentrated to as high as 50 mg/ml in 50 mM NaCl, 20 mM Tris pH8 and 1 mM DTT, with an estimated purity over 95%. The Mcm4/6/7 hexamer we obtained from *E. coli* showed helicase activity on forked dsDNA substrate, which was made by anneal a labeled ssDNA to the circular M13 ssDNA (Figure 6C). The helicase activity of this hexamer was comparable to the Mcm4/6/7 we purified from insect cells.

As shown in Figure 3 and Additional file 1: Figure S1, the nFL fragment of Mcm6 expressed for this Mcm4/6/7 hexamer contains a highly disordered internal loop that is close to its C-terminus, which might be a problem for future crystallographic studies. Thus a nFL fragment of Mcm6 without that disordered loop was used in the co-expression and copurification. A hexamer peak still appeared but the yield of the hexamer is much lower (Figure 6Ae), indicating the putatively disordered part may contribute to stabilizing the hexamer.

## Discussion

Eukaryotic MCM proteins can form various complexes including dimers, trimers, tetramers, hexamers and double-hexamers. In addition to interactions between different subunits, self-interactions of some MCM proteins have also been shown [7,23]. Most of those studies performed yeast two-hybrid assays and co-immunoprecipitation (co-IP) to investigate and demonstrate the interactions, and there are some disagreement of MCM protein interaction pairs in the literature [24,25]. Gel filtrations have been used to study interactions among *Saccharomyces cerevisiae* MCM proteins (scMCM) [26], in which all full-length scMCM proteins (except scMcm5) form large aggregates, implying folding problems of full-length MCM proteins, especially when expressed individually.

In this study, we expressed and purified a series of Mcm 4, 6 and 7 fragments as a way to investigate domain structures, folding, and roles in oligomerization. At the same time, we have obtained a soluble, stable and functional complex of Mcm4/6/7 from *E. coli*, potentially useful for future structural and functional studies.

### Oligomeric states of N-terminal fragments of Mcm4, 6 and 7

High-resolution structural data were available from the N-terminal fragments of MtMCM and SsoMCM, which forms head-to-head double hexamers [18] or single hexamer [22]. In addition to sequence similarity, several features in this partial MtMCM structure are also shown for MCM proteins in eukaryotes. First, a zinc-finger motif is crucial in mediating hexamer-hexamer interaction. Putative zinc-finger motifs are also found on Mcm4, 6 and 7 (Figure 2A), which are defined by C(X)_2_C(X)_18_C(X)_2_C. The biochemical importance of this motif has been shown by mutagenesis studies on archaeal and eukaryotic MCM proteins [27-29]. Second, the N-termini of MCM proteins play important roles in hexamer formation as well, which were shown by the deletion of 204 amino acid residues at N-terminus spMcm2 [30]. Furthermore, the N-terminals of an archaeal MCM are also shown to stimulate helicase activity of C-terminals [31].

One question to be investigated in this study is if the N-terminal domains of eukaryotic MCM2-7 also play the same structural role in hexamerization. According to the structural prediction (Figure 2B), both Mcm4 and Mcm6 have very disordered N-termini. It was noted previously that three yeast MCM proteins, Mcm2, 4 and 6, have extended N-termini when compared to the other MCM proteins (Additional file 1: Figure S1) [32]. These extended N-termini are rich in serine and threonine residues and was reported to play a redundant role in initiation of DNA replication through phosphorylation [33]. Unlike the N-terminal domains of MtMCM and SsoMCM, which can form stable hexamers, no strong inter-subunit interactions were identified of the N-terminal domains of spMCM [18,22]. However, we found the extended N-terminus, the first 62 amino acid residues on Mcm6, is required for self-interaction, as deleting the 62 amino acid residues shifted the dimer to the monomer peak (Figure 4Aa-f). Self-interactions of Mcm6 have been demonstrated by yeast two-hybrid assays, co-IP and gel filtration, even though unclear about the oligomeric states [23,24,26,34,35]. Even though the 62 amino acid residues were required for dimerization of N-terminal fragments of Mcm6, an nFL Mcm6, 6 F9, formed Mcm4/6/7 hexamers (Figure 6Ad). This result suggests the extended N-terminus of Mcm6 is only involved in the interactions between two N-terminal fragments. Furthermore, unlike the extended N-termini found on Mcm2 and 4 in all eukaryotic organisms (Additional file 1: Figure S1), the extended N-termini of Mcm6 only exists in *S. cerevisiae* and *S. pombe*, suggesting that the roles associated with the extended N-termini of Mcm6 may only be restricted to yeast.

For the N-terminal fragments of Mcm7, we observed two elution peaks in the gel filtration profile that were in agreement with dimers and monomers (Figure 4Ag and j). Self-interactions of Mcm7 were previously reported [23,24,26,35]. Our observation that the N-terminal fragment of Mcm7 form dimers may suggest their potential involvement in the self-association of Mcm7. Unlike weak self-interactions of Mcm7 reported previously, the two oligomeric states of the N-terminal fragment can be separated by ion-exchange chromatography (Figure 4Ah and i), which indicated a relatively strong interactions between the two N-terminal fragments.

In contrast to Mcm6 and 7, the extended N-terminus of spMcm4 is not likely to play a role for intersubunit interactions. No self-interactions of N-terminal fragments of Mcm4 were identified.

### Oligomeric states of core fragments of Mcm4, 6 and 7

The MCM core is conserved in MCM proteins from archaea to human (Additional file 1: Figure S1) [32,36]. As shown in Figure 4A, the MCM core contains there ATPase consensus motifs, the Walker A motif, the Walker B motif and the Arg-finger motif. Mutagenesis has been done on the putative ATP binding site in this region to prove the importance of this region in modulating oligomerization of MCM proteins [21,30,37]. However, possibly due to the lack of structurally important zinc finger motifs, core fragments alone have been reported incapable to oligomerize by themselves [31]. In our study, a core fragment of Mcm4, 4C1, formed two oligomeric forms consistent with hexamers and 12-mers (Figure 5c and b). Given the fact that the zinc finger motifs were required for head-to-head double-hexamerization of the MtMCM [18], the 12-mers we identified here are not likely to be the head-to-head double hexamers.

### Oligomeric states of individually expressed near-full-length Mcm4, 6 and 7

Most full length eukaryotic MCM proteins have been reported to form aggregates when expressed individually [26]. In this report, we also found that most expressed nFL fragments of Mcm4, 6 and 7 formed aggregates or and did not behave well in solution. Nonetheless, one nFL fragment of Mcm7 was found to be soluble and form two oligomeric states. As shown in Figure 5e, a Mcm7 construct with His tag, 7 F4, elute in gel filtration chromatography as a monomer peak and a peak of about 1000 kDa (equivalent to a 12-mer) (Figure 5f). The ~1000 kDa complex of 7 F4 seemed to be quite stable and sensitive to neither salt concentration nor protein concentration. Unlike the core fragment of Mcm4 we reported, which showed a large complex peak on gel filtration as well, this nFL fragment of Mcm7 contains all key elements for oligomerization of MCM proteins, and the large complex observed here likely is a double-hexamer.

When the same Mcm7 construct, 7 F4, was fused to GST at its N-terminus, aggregates and monomeric peaks on gel filtration were observed, which is different from the behavior of His-7 F4. This result indicates the usage of different tags fused even to the same end can have a different effect on protein oligomerization.

Surprisingly, no dimer of the 7 F4 fragment was observed, though the N-terminal fragments of Mcm7 were capable of dimerization. One explanation might be that the addition of the MCM core region on the nFL fragment further strengthens the protein’s capability to oligomerize, resulting in a cooperative shift from dimeric state to a higher oligomeric state. On the other hand, if the dimer interfaces of the N-terminal fragments are head-to-head instead of side-to-side, the interfaces may not be strong enough to overcome the entropy increase of the much longer molecule as formed when the fragments are long enough to include the MCM core region. This may also explain why the longest N-terminal fragment of Mcm6, 6 N3, was only found in monomeric state (Figure 4Ae).

### Co-expression of Mcm4/6/7 and purification of the soluble complex

Both Mcm2-7 hexamers and Mcm4/6/7 hexamers were co-expressed and copurified from the same host cell cultures as reported before [23,38]. Individually expressed MCM proteins tend to aggregate, especially when expressed in *E. coli*[26]. *In vivo*, it has been evaluated that MCM proteins are very abundant in cells and expression level of most MCM proteins are very stable through the cell cycle [4,7]. Some sub-complexes of MCM proteins were also identified by *in vivo* cross-linking [24].

We used a polycistronic strategy to achieve co-expression of the fragments of Mcm4, 6 and 7 (Figure 1A). It should be noted that the success of the polycistronic expression is highly dependent on the sequence around the ribosome binding sites (RBS), known as Shine-Dalgarno sequence in *E. coli*[39,40]. Which explains why we were unable to have some ORFs expressed, such as 6 N2, 6 N2-His and 4 F5 (Figure 1B).

Both Ni-NTA and glutathione resin was used to pull down the tagged fragments. If two fragments bind each other strongly, non-tagged or otherwise tagged fragments would be co-pulled down. However, unfolded or misfolded proteins often aggregate together on resin, leading to false positive results. Thus, we analyzed elution instead of protein-bound resin by SDS-PAGE to elucidate the binding pairs. Co-expression results were compared to co-lysis results to demonstrate that some nFL fragments have to be co-expressed to fold properly (Figure 1B *Asterisk*).

As shown in Figure 6Ad and 1Dd, the Mcm4/6/7 hexamer composed of the nFL fragments was obtained from co-expression in *E. coli*. Helicase assay with this hexamer was carried out and showed an activity comparable to that of the Mcm4/6/7 hexamer we purified from insect cells before. The yield (10 mg/12 L culture) and purity (over 95% purity) obtained using this *E. coli* co-expression provide a system for future structural and functional studies of this MCM sub-complex.

**Figure 7:**
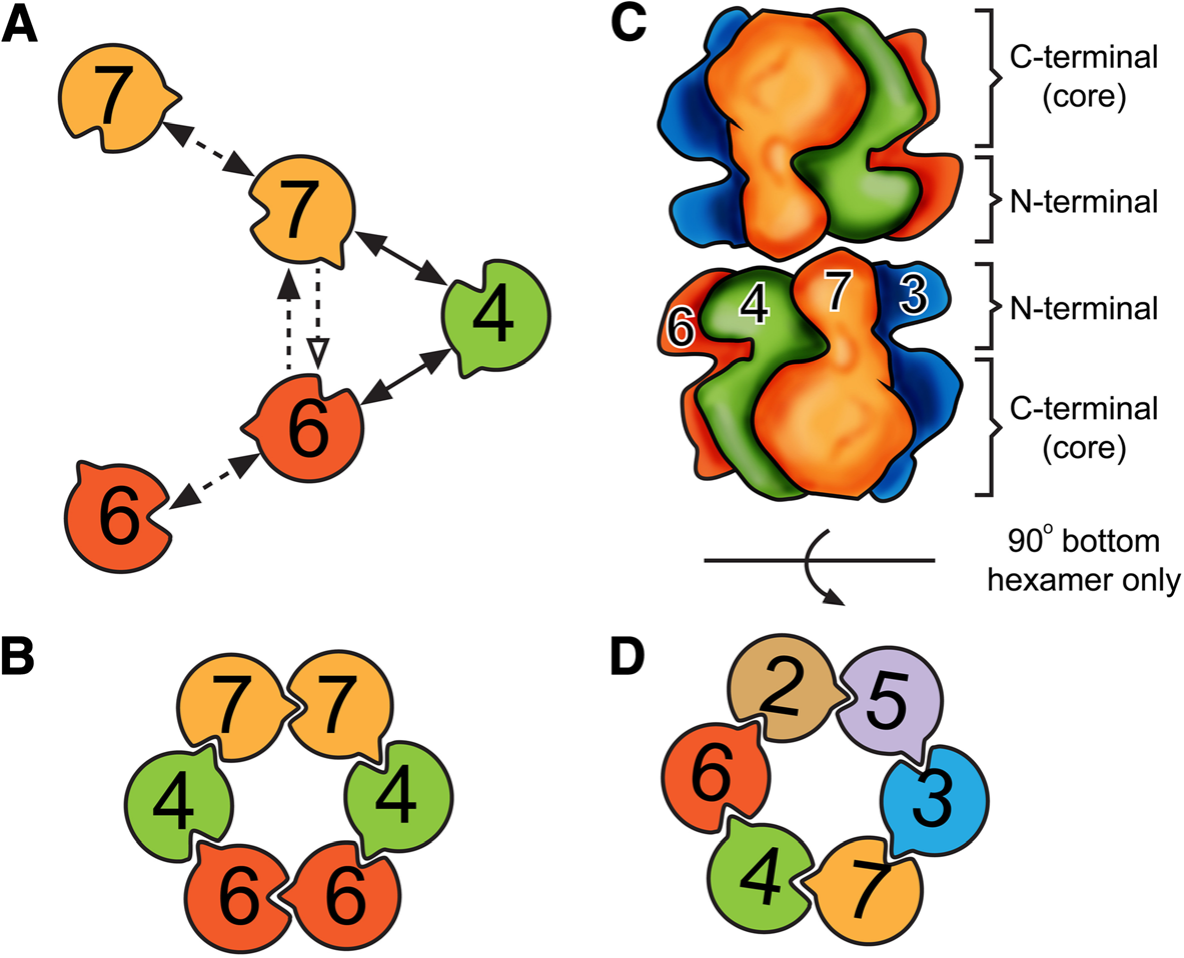
**Schematics of proposed models of the Mcm4/6/7 hexamer and the Mcm2-7 double-hexamer.** (**A**) Summary of interactions identified in this report. Double arrows: reciprocal interactions. Single arrows: unidirectional interactions. Hallow arrows: weak interactions. Solid lines: stable homogeneous oligomeric states, such as dimers. Dashed lines: heterogeneous oligomeric states: such as aggregates. (**B**) Model of the Mcm4/6/7 hexamer. This model is based on the interactions identified in Panel **A**, which is consistent with the model proposed previouslyfor *S. cerevisiae* MCM [26], and human MCM [23,24,42]. (**C-D**) Model of hexamer-hexamer interactions for the Mcm2-7 double-hexamer. This model is based on Figure 1A of [6], showing a proposed Mcm7/7 interaction that locks the orientation of two hexamers. The convex and the concave on each subunit in this figure represent the P-loop of the Walker A motif and the Arg-finger motif, respectively.

A summary of binding pairs identified by our results was illustrated as in Figure 7A. The Mcm4/Mcm6 dimer and the Mcm4/Mcm7 dimer were identified and characterized by both gel filtration and SDS-PAGE in this study (Figure 6Ba and b). We also showed self-interactions of Mcm7 and Mcm6, especially in the case of 7 F4, which formed a large complex that might be a double-hexamer. These results are consistent with previous reports [23,24,26,35,41]. Our data support the arrangement model of the Mcm4/6/7 hexamer for the six subunits of spMCM (Figure 7B) that was proposed for *S. cerevisiae* MCM [26], and human MCM [23,24,42]. An alternative arrangement model, in which binding between Mcm6 and Mcm7 occurs, was proposed in a previous report [43]. The literatures have some disagreement about the interactions between Mcm6 and Mcm7. Evidence showing no direct binding [24], or weak binding [23], or strong binding [35] of Mcm6/7 pair was previously reported from different groups. Our results showed a strong binding of Mcm6 and Mcm7 by affinity pull down from co-expression (Figure 1B). However, no stable Mcm6/7 dimer was present on gel filtration analysis (Figure 6Bc). It should be noted even though there is no direct contact between Mcm6 and Mcm7 in the proposed planar ring-shaped hexamer structures, contact between them might exist in a staggered globular shaped structure in which each MCM subunit has direct contact with at least four subunits [44]. This staggered globular shaped Mcm2-7 hexamer is composed of two layers of trimmers and was only reported for *S. pombe*, but not for human. It is likely the strong interaction between Mcm6 and 7 is unique to *S. pombe* and contributes to the formation of the globular hexamer.

As for the N-terminal fragments of Mcm4, 6 and 7, we did not identify any binding pairs between different subunits, even in co-expression (Figure 1B). But we observed stable homo-dimers of N-terminal fragments of Mcm6 and 7 (Figure 4Aa, c, g and j). Given the strong structural evidence for double-hexamers from MtMCM and scMCM [5,6,18,45], the interfaces of these dimers are likely to be head-to-head. The 3D reconstruction model processed from C2 point group symmetry clearly showed the two MCM hexamers are connected by head-to-head “protein bridges” [6]. Based on the 30 Å 3D EM reconstruction model, we proposed a Mcm2-7 double-hexamer model as illustrated in Figure 7C. The head-to-head interactions between two identical subunits from each hexamer can only occur at most twice in the hexamer-hexamer interface, and all other interactions should be between different MCM subunits. We observed homo-dimer only for the N-terminal fragment of Mcm6 and 7. Because the 62 amino acid residues extended N-terminus required for Mcm6 dimerization is only found in yeast, the N-terminus-to-N-terminus interactions between two identical subunits should be through Mcm7. In our model, the orientation between two hexamers is locked by the specific interaction between two Mcm7 subunits, and double-hexamerization is further stabilized by nonspecific interactions between zinc finger domains of the other Mcm subunits.

## Conclusions

Here we described a systematic characterization of the biochemical properties of different domains of *S. pombe* Mcm4, 6 and 7 using *E. coli* expression. The oligomeric states and inter-subunit interactions have been determined with purified protein *in vitro*. A co-expression strategy was also developed to obtain large amount of soluble, stable and functional Mcm4/6/7 hexamer complex from *E. coli*, which can be useful for future structural and biochemical studies. Based on our results and the literature, we suggest an arrangement model of *S. pombe* Mcm4/6/7 hexamer and the hexamer-hexamer interactions in the Mcm2-7 double-hexamer.

## Abbreviations

Mcm: Minichromosome maintenance; S. pombe: *Schizosaccharomyces pombe*; S. cerevisiae: *Saccharomyces cerevisiae*; pre-RC: Pre-replicative complex; ORC: Origin recognition complex; Mt: *Methanothermobacter thermautotrophicus*; Sso: *Sulfolobus solfataricus*; LTag: Simian virus 40 large tumor antigen; E. coli: *Escherichiai coli*; PCR: Polymerase chain reaction; GST: Glutathione S-transferase; sp: *S. pombe*; sc: *S. cerevisiae*

## Competing interests

The authors declare that they have no competing interests.

## Authors’ contributions

MX designed, expressed and purified the protein constructs; carried out all assays for biochemical characterization. YPC provided advice for designs of truncated proteins. XSC supervised the project. All authors read and approved the final manuscript.

## Supplementary Material

Additional file 1: Figure S1 Sequence alignment of MCM proteins from various organisms. SsoMCM, *Sulfolobus solfataricus* MCM. MtMCM, *Methanothermobacter thermautotrophicus* MCM. This result was generated by ClustalX as described under “Methods”. Click here for file

Additional file 2: Figure S2 Disordered profile plots of Mcm6 and 7. The disordered profiles were generated by the DISOPRED server at University College London. Click here for file
