# Supplementary figures and images for: Expression, purification and biochemical characterization of Schizosaccharomyces pombe Mcm4, 6 and 7
Source: BMC Biochem. 2013 Feb 27;14:5. doi: 10.1186/1471-2091-14-5 (PMC3605359; doi:10.1186/1471-2091-14-5)

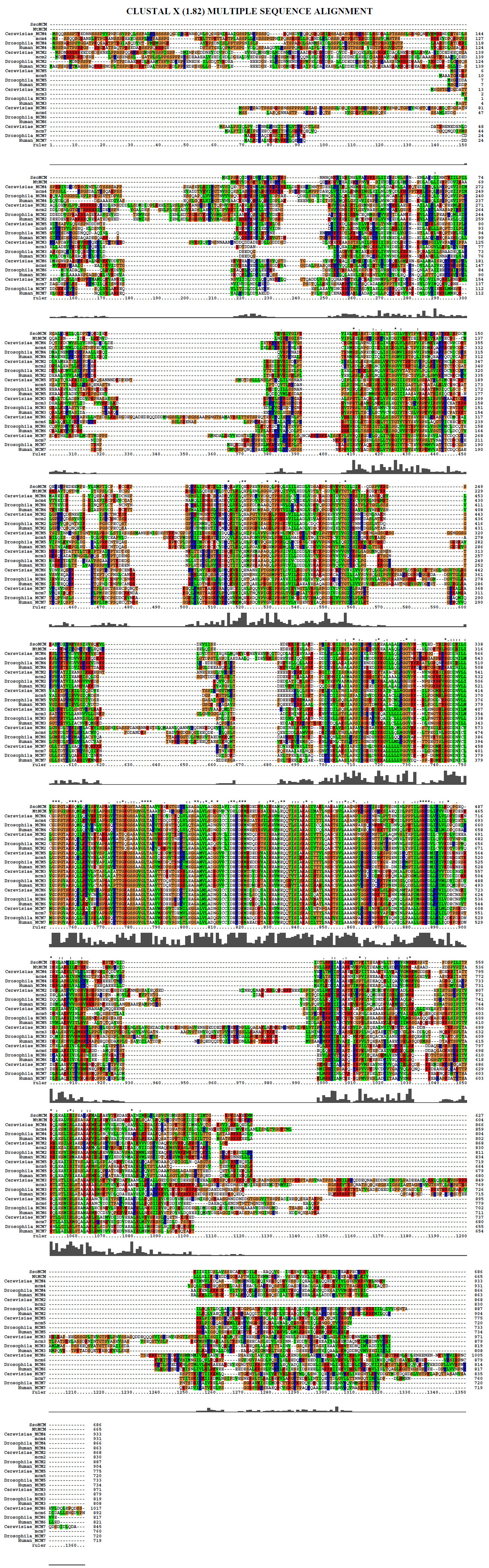

Supplement: Additional file 1: Figure S1 — Sequence alignment of MCM proteins from various organisms. SsoMCM, Sulfolobus solfataricus MCM. MtMCM, Methanothermobacter thermautotrophicus MCM. This result was generated by ClustalX as described under “Methods”. [file 1471-2091-14-5-S1.jpeg]

**A**

Disordered profile plot of Mcm6

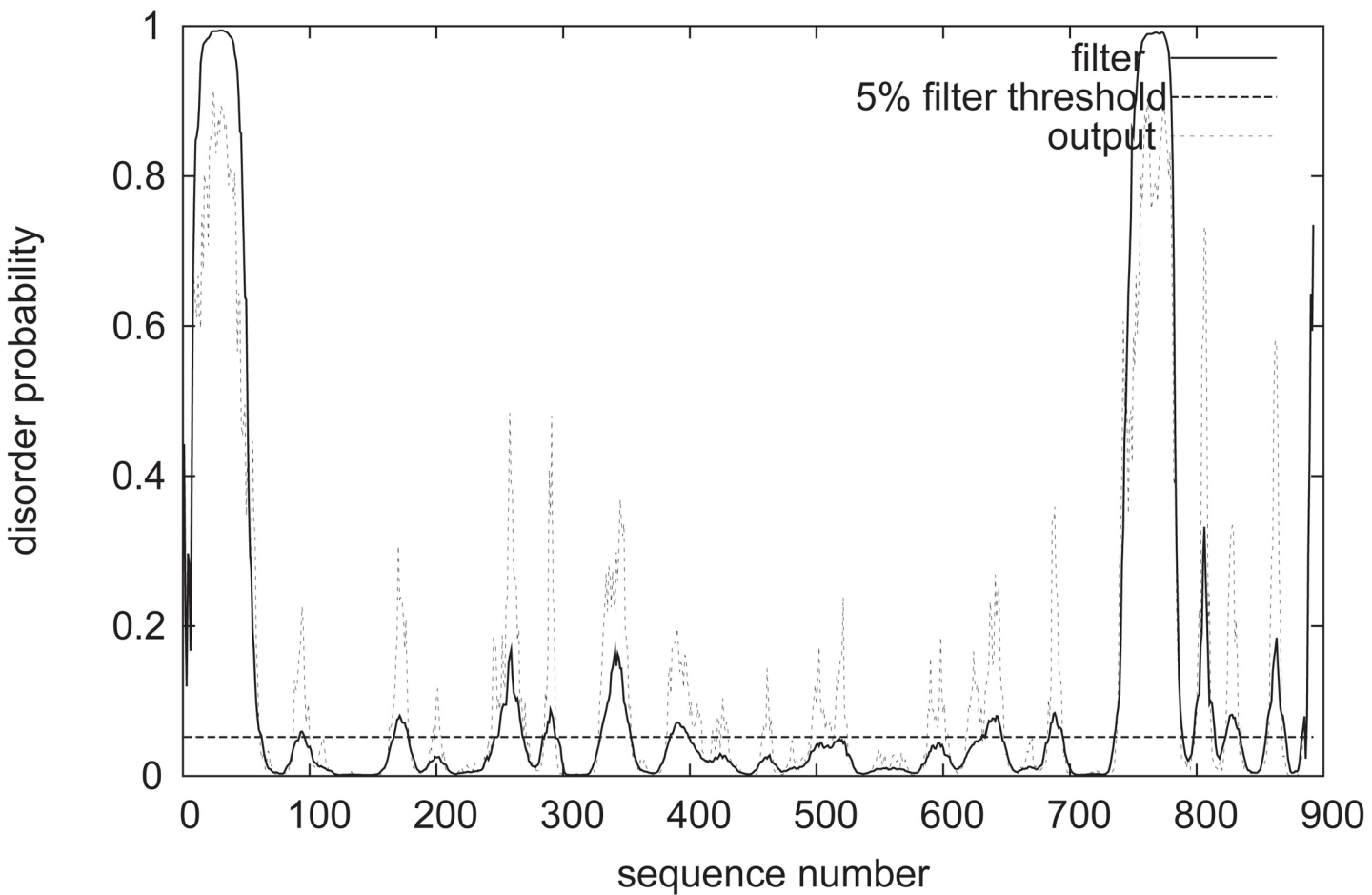**B**

Disordered profile plot of Mcm7

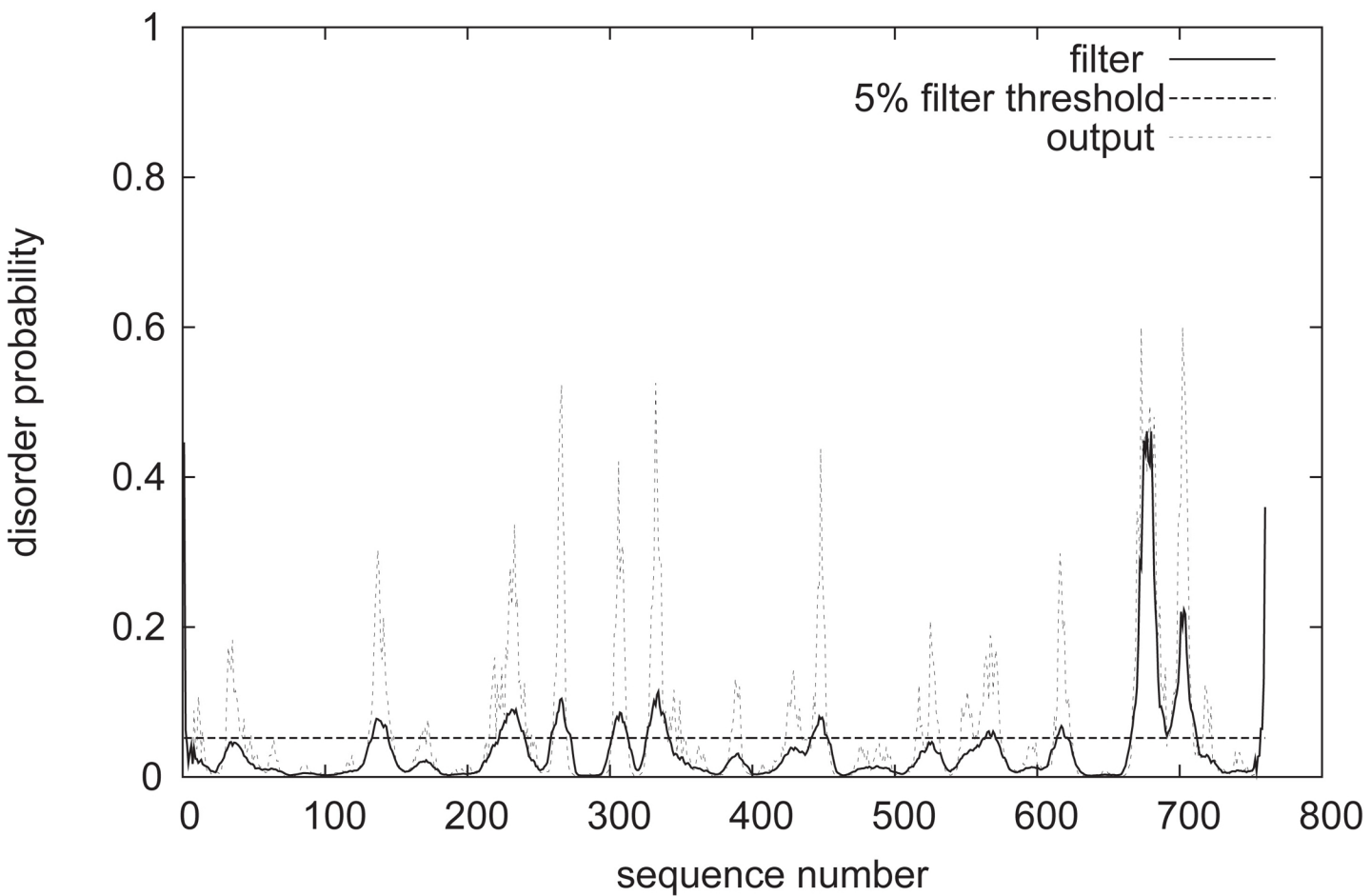

Supplement: Additional file 2: Figure S2 — Disordered profile plots of Mcm6 and 7. The disordered profiles were generated by the DISOPRED server at University College London. [file 1471-2091-14-5-S2.pdf]
